# Recent Advances in PEEK for Biomedical Applications: A Comprehensive Review of Material Properties, Processing, and Additive Manufacturing

**DOI:** 10.3390/polym17141968

**Published:** 2025-07-17

**Authors:** Samreen Dallal, Babak Eslami, Saeed Tiari

**Affiliations:** 1 Biomedical Engineering Department, Widener University, One University Place, Chester, PA 19013, USA; sadallal1@widener.edu; 2 Mechanical Engineering Department, Widener University, One University Place, Chester, PA 19013, USA; beslami@widener.edu

**Keywords:** PEEK, 3D printing, biomedical implants, surface modification, additive manufacturing

## Abstract

Polyetheretherketone (PEEK) is a high-performance thermoplastic polymer widely recognized for its distinct mechanical strength, chemical resistance, and biocompatibility. These characteristics make it suitable for a wide range of applications, particularly in medical, aerospace, chemical, and electronics fields. Conventional processing techniques, such as 3D printing, molding, and extrusion, are widely employed for PEEK fabrication. This review critically examines recent advancements in PEEK research, with an emphasis on additive manufacturing techniques that are expanding its applications in the medical field. We provide an in-depth analysis of PEEK’s intrinsic properties, diverse processing methods, and current challenges that hinder its wider adoption. In addition to evaluating PEEK’s performance, this review compares it with alternative biomaterials—such as titanium and ultra-high molecular weight polyethylene (UHMWPE)—to explore its advantages and limitations in biomedical applications. Furthermore, this review discusses cost considerations, regulatory constraints, long-term clinical performance challenges, and failure modes that are essential for validating and ensuring the reliability of PEEK in clinical use. By synthesizing the recent literature, particularly from the last decade, this review highlights the significant potential of PEEK and underscores ongoing research efforts aimed at overcoming its limitations, paving the way for its broader implementation in advanced technological applications.

## 1. Introduction

Polyetheretherketone (PEEK) is a semi-crystalline thermoplastic polymer belonging to the polyaryletherketone (PAEK) family, and is widely used in biomedical, aerospace, and industrial applications due to its exceptional mechanical, thermal, and chemical resistance properties [1]. PEEK is typically synthesized via step-growth polymerization through the nucleophilic aromatic substitution reaction between 4,4′-difluorobenzophenone and hydroquinone in the presence of a base such as potassium carbonate and high-boiling solvents like diphenyl sulfone under an inert atmosphere at temperatures ranging from 300 to 350 °C [2]. This reaction produces linear chains of PEEK with high molecular weight, resulting in its robust mechanical performance. At the molecular level, PEEK consists of repeating units of aromatic rings linked by ether and ketone groups (–C_6_H_4_–O–C_6_H_4_–O–C_6_H_4_–CO–). The rigid aromatic backbone provides high thermal stability, while ether linkages contribute to its processability [3]. PEEK exhibits a semi-crystalline structure with crystallinity typically ranging from 30% to 40%, depending on processing conditions. Its crystalline phase is orthorhombic with tightly packed chains, enhancing its chemical resistance and mechanical strength [2]. Additionally, PEEK has a glass transition temperature (Tg) of approximately 143 °C and a melting temperature (Tm) around 343 °C [2]. Its high thermal stability allows sterilization via autoclaving without degradation, making it suitable for reusable biomedical devices. The crystallization temperature ranges between 160 and 310 °C, depending on cooling rates during processing, which influences its degree of crystallinity and thus mechanical performance [4].

These properties make PEEK highly desirable in various industries, including aerospace, electronics, and particularly medicine [5]. In biomedical applications, PEEK’s elastic modulus is comparable to that of cortical bone, and resistance to physiological environments makes it a compelling alternative to traditional materials such as titanium and ultra-high molecular weight polyethylene (UHMWPE) [6]. While titanium is well-regarded for its strength and corrosion resistance, its high stiffness can lead to stress shielding and subsequent bone resorption. In contrast, PEEK offers mechanical compatibility with bone, enhancing implant integration and reducing complications [7]. These properties make PEEK comparable to bone in stiffness while providing sufficient toughness, thus making it attractive for orthopedic and spinal implants [2].

The material’s durability, thermal resistance, and lightweight nature further contribute to its suitability for orthopedic devices such as spinal cages, craniomaxillofacial prostheses, bone screws, dental implants, and joint replacements [5,8,9].

**Figure 1:**
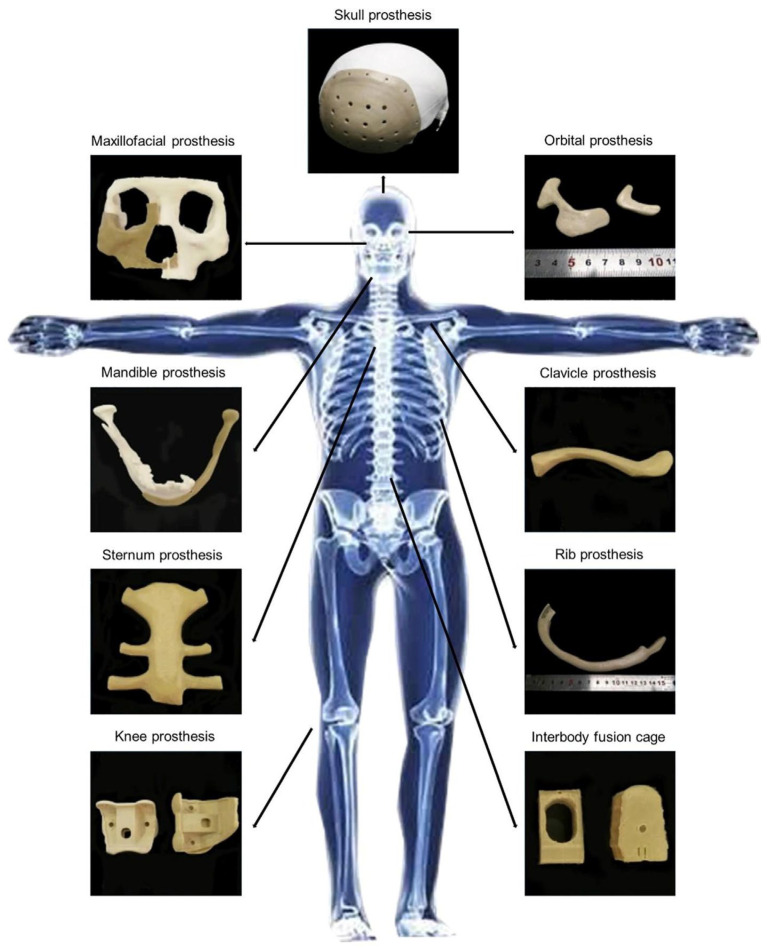
Schematic illustrating the applications of PEEK implants in orthopedics. Reprinted from Ma et al. [10]. Licensed under CC BY-NC-ND 4.0 (https://www.sciencedirect.com/science/article/pii/S1878535220305384#f0005 (accessed on 13 July 2025)).

Overall, the combination of excellent thermal resistance, mechanical performance, chemical inertness, and biocompatibility makes PEEK an attractive material for biomedical applications, especially where metallic implants are limited by weight, imaging artifacts, or lack of tailored properties. Figure 1 illustrates examples of PEEK applications in orthopedic implants [10].

PEEK can be processed via several manufacturing techniques, each offering distinct advantages depending on the application. Injection molding is suitable for the mass production of components with complex geometries due to its high melt strength and thermal stability. Extrusion is commonly used to produce rods, sheets, and filaments for machining or additive manufacturing. Subtractive Machining, including the milling and turning of extruded PEEK rods, enables the fabrication of high-precision implant geometries. While these conventional methods remain prevalent in industry, they offer limited flexibly in controlling key structural features such as porosity and surface roughness [11,12,13].

In recent years, additive manufacturing (AM), particularly three-dimensional (3D) printing, has emerged as a transformative approach, enabling the creation of customized implants with tailored characteristics, including surface texture, porosity, and mechanical strength [9,14]. Among AM techniques, fused deposition modeling (FDM)—also known as fused filament fabrication (FFF)—is the most widely used for polymer fabrication, owing to its low maintenance costs, material versatility, and accessibility for consumers [15,16]. Leveraging computer-aided design (CAD) and computer-aided manufacturing (CAM) technologies, PEEK implants can be precisely tailored to meet individual clinical requirements [17].

Overall, the combination of excellent thermal resistance, mechanical performance, chemical inertness, and biocompatibility makes PEEK an attractive material for biomedical applications, especially where metallic implants are limited by weight, imaging artifacts, or a lack of tailored properties. However, while significant efforts have been made to enhance the mechanical properties of polymers commonly used in FDM—such as polylactic acid (PLA), acrylonitrile butadiene styrene (ABS), and polyethylene terephthalate glycol (PETG)—further research is still needed to fully optimize the mechanical performance of PEEK [18]. This paper aims to review recent advancements in PEEK research, with a particular focus on biomedical applications and the identification of current knowledge gaps.

## 2. Processing Techniques

### 2.1. Traditional Methods

As demand for PEEK continues to grow, additive manufacturing—particularly three-dimensional (3D) printing fabrication techniques—have been used to process this high-performance polymer. However, before the emergence of additive manufacturing, PEEK components for biomedical and industrial applications were primarily fabricated using conventional manufacturing techniques such as injection molding, compression molding, extrusion, and subtractive machining (milling and turning) [19,20,21]. Injection and compression molding are widely used for the mass production of PEEK components due to their high efficiency and capability to produce complex geometries with excellent dimensional accuracy and surface finish. Medical-grade PEEK can be injection molded into implants and device components; however, precise temperature control is essential due to its high melting temperature (~343 °C) and processing temperature range (350–400 °C) [2]. Special attention is also required for mold design and cooling to minimize residual stresses and warpage. Additionally, injection molding is commonly used to manufacture lightweight PEEK components for aerospace applications, offering energy efficiency and design flexibility [22,23]. Compression molding can also be combined with salt beads as porogens to fabricate porous PEEK structures, which demonstrates the feasibility of tailoring pore size for orthopedic applications [24].

Extrusion is another common method to produce PEEK in the form of rods, sheets, or filaments. Extruded PEEK rods are often used as stock materials for further machining into final implant geometries [25]. On the other hand, subtractive machining techniques such as milling, turning, and drilling are frequently employed to produce PEEK implants with high dimensional tolerances. PEEK’s machinability is considered favorable compared to metals due to its lower hardness and density, allowing for easier cutting with reduced tool wear. However, thermal management is critical during machining because excessive heat buildup can lead to local melting, surface damage, or dimensional inaccuracies [25].

Overall, conventional manufacturing techniques remain important for the production of standardized, mass-produced PEEK medical components. However, they are limited in producing patient-specific or highly complex porous structures without extensive tooling and secondary operations, a gap now addressed by additive manufacturing technologies. Additive manufacturing enables the fabrication of complex geometries, minimizes material waste, and supports the creation of customizable implants at lower production costs [26]. The following section explores the application of 3D printing in biomedical applications, with a focus on its impact on implant mechanical and biological performance of implants.

### 2.2. 3D Printing

Among the various fabrication techniques available for PEEK, this review focuses on 3D printing due to its growing relevance in biomedical applications. Additive manufacturing methods such as fused deposition modeling (FDM) and selective laser sintering (SLS) enable the layer-by-layer construction of customized implants. FDM, in particular, is more widely adopted because of its lower cost, simpler setup, and ease of operation [27]. Additionally, the compact size of most PEEK-compatible FDM printers makes them suitable for clinical and laboratory environments.

However, PEEK’s high melting point—requiring nozzle temperatures exceeding 340 °C—limits its compatibility with standard 3D printers. Unlike common polymers such as PLA, which print at 190–230 °C, PEEK demands specialized high-temperature printers [28]. These extreme conditions can lead to warping, shrinkage, delamination, and poor interlayer adhesion, all of which compromise mechanical performance [29,30]. To address these challenges, researchers have explored thermal process control, including precise adjustments of nozzle and chamber temperatures [31]. For example, Liu et al. (2024) printed PEEK specimens at 420 °C and applied post-print heat treatment at 300 °C for two hours, which improved both crystallinity and interlayer adhesion [32]. (Further discussion on crystallinity is provided in Section 4.3).

In another study, Xiaoyong et al. (2017) [28] printed 1BA-type tensile specimens with high precision using a 0.2 mm layer height, 0.8 mm shell thickness, and a 20 mm/s print speed. They varied bed and ambient temperatures to evaluate their effects on tensile strength. The highest mechanical performance was achieved with a 130 °C bed and 60 °C ambient temperature, highlighting the critical role of thermal conditions in producing high-strength PEEK components.

Beyond thermal parameters, mechanical properties are also influenced by print settings such as layer height and speed. Chithambaram and Senthilnathan (2024) demonstrated that a 0.15 mm layer height and a print speed of 20 mm/s optimized hardness and wear resistance in FFF-printed PEEK [33].

**Figure 2:**
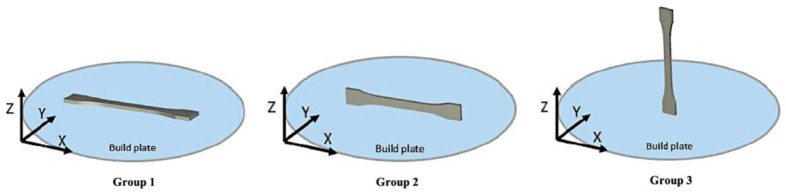
Dog bone samples printed in different orientations. Adapted from “Effect of Printing Parameters on Mechanical Performance of Material-Extrusion 3D-Printed PEEK Specimens at the Point-of-Care,” by Zarean et al. [14]. Licensed under CC BY 4.0 (https://www.mdpi.com/2076-3417/13/3/1230 (accessed on 13 July 2025)).

In contrast, non-thermal parameters such as build orientation and sample position appear to have a limited impact. Zarean et al. (2023) [14] evaluated mechanical performance across specimens printed in various orientations (XX, XZ, ZX) and positions. While overall strength remained consistent, the ZX orientation exhibited slightly lower tensile strength. Figure 2 illustrates the sample orientations evaluated.

## 3. Applications

PEEK finds application across multiple engineering domains, including biomedical, chemical, electrical, and mechanical fields, owing to its exceptional thermal stability, chemical inertness, radiation resistance, and semi-crystalline structure. In electrical engineering, it is used in components such as electrodes, printed circuit boards, fire shields, and dielectric films for wafer holders [19,34]. It is also used for plasma coatings on metal surfaces to enhance corrosion resistance, wear protection, and thermal insulation [35]. However, among all its applications, PEEK has seen particularly rapid growth in the biomedical field. The following subsections explore its evolving role in medical implants, followed by highlights of its continued use in other industries.

### 3.1. PEEK in Orthopedics Applications

PEEK’s excellent biocompatibility and mechanical properties have made it an increasingly successful option as an orthopedic implant biomaterial. Since receiving approval from the U.S. Food and Drug Administration (FDA) in 1998, PEEK has been recognized as a viable polymer for bone substitution [36]. It is now widely accepted as a radiolucent alternative to metallic biomaterials, and extensive research is being conducted to improve its physical and mechanical properties for various orthopedic applications [2]. Below are some key orthopedic areas where PEEK has been successfully utilized.

#### 3.1.1. Craniomaxillofacial Reconstruction

Craniomaxillofacial (CMF) trauma resulting from sports injuries, accidents, disease, or tumor resections often requires complex reconstructive surgery [37,38]. A widely adopted approach involves the use of computer-aided design (CAD) and computer-aided manufacturing (CAM) technologies in conjunction with 3D printing to produce patient-specific implants. While metals, polymers, and ceramics have traditionally been utilized, PEEK has gained increasing attention due to its favorable mechanical properties, biocompatibility, and thermal stability—features that permit sterilization without material degradation [38]. These characteristics make PEEK particularly well-suited for craniofacial implants and cranioplasty procedures, which aim to reconstruct skull defects using synthetic or biological materials [39].

Compared to traditional autografts or allografts, synthetic materials such as PEEK offer reduced operative time and improved cosmetic outcomes [40]. PEEK plates are lightweight, flexible, cost-effective, and easily customized [41]. Moreover, its mechanical compatibility with human bone and energy-absorbing elasticity provide superior protection to brain tissue compared to titanium implants [42]. The accessibility of 3D printing has further accelerated the production of custom cranial implants, driving ongoing research into enhancing PEEK’s performance in CMF applications [43].

Despite these advantages, PEEK still faces notable challenges, including poor osseointegration and weak interfacial adhesion, which can contribute to implant loosening or long-term failure. Surface modification techniques—such as hydroxyapatite coatings—have shown promise in improving bone integration, although their long-term stability and effectiveness remain areas of active investigation [44]. Future studies should also explore the performance of PEEK implants under dynamic physiological stresses to better understand their behavior in post-operative conditions.

#### 3.1.2. Spinal Implants

PEEK applications in spinal implants have also been extensively investigated. Basgul et al. (2018) demonstrated the use of 3D-printed PEEK cages in lumbar fusion, highlighting the material’s role in maintaining implant strength [45]. A subsequent study explored FFF-printed porous PEEK cages, focusing on the influence of printing parameters—nozzle size, print speed, layer thickness, and the number of cages per build—on thermal conditions during printing. The findings showed that optimizing these parameters reduced thermal gradient, improving interlayer bonding and mechanical performance [46].

**Figure 3:**
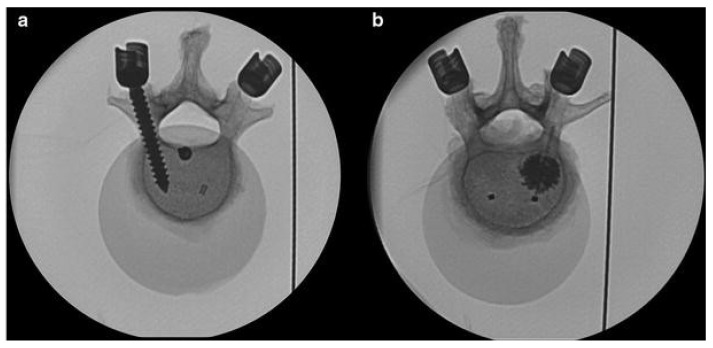
Axial fluoroscopic images of instrumented vertebrae. (**a**) CF/PEEK pedicle screws (right) and titanium control screw (left); (**b**) PMMA-augmented (right) and nonaugmented CF/PEEK (left) pedicle screws. Adapted from Lindtner et al. [47]. Licensed under CC BY 4.0 (https://link.springer.com/article/10.1007/s00586-018-5538-8 (accessed on 13 July 2025)).

Beyond optimizing printing parameters, PEEK can be reinforced with other materials such as carbon fiber to enhance implant properties. Lindtner et al. (2018) [47] investigated nonmetallic carbon fiber-reinforced PEEK (CF/PEEK) pedicle screws for spinal stabilization, comparing them to traditional titanium screws. The results showed that CF/PEEK screws performed comparably under cyclic loading until loosening, with screw stability further improved by the addition of PMMA. Additionally, the radiolucent nature of CF/PEEK minimizes imaging artifacts, offering a key advantage over metallic implants, as shown in Figure 3.

#### 3.1.3. Joint Replacement

Polyethylene (PE) is widely used in knee replacement implants; however, wear particle generation remains a leading cause of revision surgeries [48]. Consequently, interest has shifted toward PEEK and its composites as alternatives materials [49]. Scholes & Unsworth (2009) evaluated the wear characteristics of PEEK and carbon fiber-reinforced PEEK (CFR-PEEK) using a multi-directional pin-on-plate machine with low carbon (LC) and high carbon (HC) CoCrMo alloy plates, finding that CFR-PEEK offers favorable wear performance and biocompatibility compared to UHMWPE [50].

A more recent controlled trial compared PEEK and traditional CoCrMo tibial prostheses, demonstrating ideal cement penetrations and no additional complications [51]. While PEEK shows significant promise, further research is needed to validate its long-term clinical performance in knee arthroplasty.

PEEK’s favorable mechanical properties, chemical resistance, and biocompatibility have also promoted its use in hip replacement applications. Early work showed that PEEK composites are viable bearing surfaces for acetabular cups, offering performance comparable to UHMWPE [52]. Recent advances incorporated reduced graphene oxide (rGO) and calcium hydroxyapatite (cHAp) into 3D-printed PEEK structures, with optimized lattice designs to enhance mechanical performance and biocompatibility. The results emphasized the critical role of porosity and lattice architecture in replicating the mechanical behavior of natural bone [53].

In another study, a comprehensive computational wear stimulation of cobalt chromium (CoCr) on CFR-PEEK bearing couples under gait cycle demonstrated superior wear performance of CoCr-on-CFR-PEEK compared to CoCr-on-cross-linked polyethylene (XLPE) in total hip replacement. However, CoCr-on-CFR-PEEK produced a higher release of metallic particles, raising greater biocompatibility concerns than polyethylene wear [54]. Further research is needed to minimize CoCr wear rate when paired with PEEK-based bearing surfaces.

#### 3.1.4. Rib Prostheses

Custom-made PEEK rib prostheses have recently been utilized in chest wall reconstruction for conditions such as malignant tumors and congenital deformities [55]. In one study, a custom-designed rib prosthesis fabricated using FDM technology demonstrated mechanical behavior and stability comparable to natural human ribs [56]. Further research suggested that adjusting parameter combinations can enhance the mechanical properties of PEEK prostheses, providing flexibility to tailor design and strength based on specific prostheses requirements [57]. However, additional studies are needed to understand the correlation between prosthesis geometries, printing parameters, and mechanical properties.

In conclusion, PEEK and its composites demonstrate strong potential for orthopedic implant applications, though further research is needed to fully optimize their performance. Ongoing advancements continue to position PEEK as an emerging alternative to traditional biomaterials, with anticipated growth across various sectors.

### 3.2. PEEK in Dental Implants

In addition to orthopedic applications, PEEK is gaining attention in dentistry due to its numerous advantages over traditional dental implant materials. It has been utilized in removable dentures, maxillofacial prostheses, and crowns owing to its low elastic modulus, high radiation resistance, excellent thermal stability, and insolubility in oral solvents [58]. PEEK’s elastic modulus (approximately 3–4 GPa) is closer to that of cortical bone (14 GPa) compared to metals like titanium (approximately 102–110 GPa), reducing stress shielding—a mechanical phenomenon in which extensive stiffness surrounding the implant leads to bone resorptions [59,60]. Furthermore, PEEK’s radiolucent nature results in fewer artifacts in magnetic resonance imaging (MRI) compared to metallic materials such as titanium [61].

**Figure 4:**
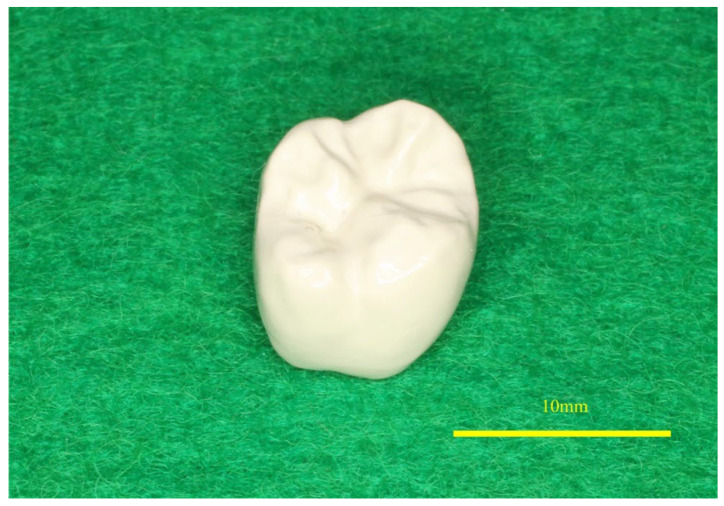
The occlusal surface of the PEEK crown. Adapted from Kimura et al. [62]. Licensed under CC BY 4.0 (https://www.nature.com/articles/s41598-022-23458-5 (accessed on 13 July 2025)).

Kimura et al. (2022) [62] conducted an in vivo study of PEEK crowns placed on the molars of nineteen subjects, assessing the condition of the crowns at cementation and after six months to evaluate patient satisfaction, masticatory ability, and occlusal force. Figure 4 illustrates the fabricated PEEK crown. The results showed no fractures, cracks, or detachment, and no additional interventions were required. Patient satisfaction was high, with complaints mainly related to mastication and esthetics. Occlusal pressure and force remained within the standard range between treated and untreated sides. In a more recent study, Yoshida et al. (2025) [63] hypothesized that PEEK implants would reduce stress shielding and promote bone modification compared to Ti implants. Mechanical testing on artificial bone surfaces proved that PEEK implants transmit more stress than Ti implants, reducing bone resorptions and stress shielding.

However, unmodified PEEK can be challenging in dental applications due to its bioinert nature and hydrophobicity. To address these limitations, research has shifted toward the development of modified PEEK. Najeeb et al. (2016) reviewed several nano-modification strategies, including the incorporation of bioactive apatite and the application of synthetic osteoconductive hydroxyapatite, to improve PEEK’s performance and bioactivity [64]. Additionally, surface modification techniques —such as plasma coating, chemical treatment, and physical treatment—have been shown to significantly enhance PEEK’s mechanical and biologic properties [65]. Further details on surface modification techniques are discussed in Section 5.1.

### 3.3. Other PEEK Applications

**Figure 5:**
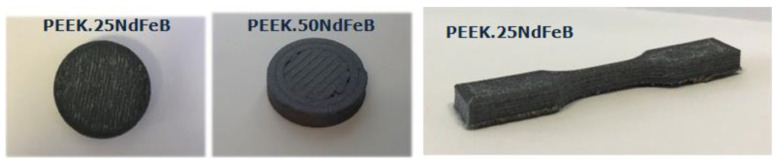
Three-dimensionally printed magnetic sample of PEEK NdFeB 25 (left), PEEK NdFeB 50 (center), and tensile test sample PEEK NdFeB25. Adapted from Pigliaru et al. [67]. Licensed under CC BY 4.0 (https://jmscomposites.springeropen.com/articles/10.1186/s42252-020-00006-w (accessed on 13 July 2025)).

Beyond healthcare applications, PEEK’s unique properties have enabled its use across a wide range of industries. In aerospace, PEEK has gained attention for its chemical resistance and thermal stability [66]. Permanent rare earth magnets, such as neodymium–iron–boron (NdFeB), are currently used in space mechanisms and equipment—such as drive systems, motors, and electronic components—for their high magnetic performance and low volume and weight. However, their poor corrosion resistance makes them susceptible to degradation of magnetic properties. To address this issue, Pigliaru et al. (2020) [67] developed a PEEK-NdFeB composite filament to protect the magnetic alloy from corrosion. Figure 5 illustrates the printed samples of PEEK-NdFeB composites.

Carbon fiber-reinforced PEEK (CF/PEEK) filaments have shown promise in radar detection technologies and electromagnetic wave (EMW) absorption due to their outstanding mechanical and electrical properties. Zhang et al. (2025) reported that CF/PEEK-based pyramidal honeycomb metastructures exhibit superior mechanical performance alongside enhanced EMW absorption capabilities [68]. In the field of electronics, CF/PEEK composites have demonstrated improved electrical conductivity and thermal performance. For instance, Martin et al. (2024) [69] utilized CF/PEEK facesheets in the fabrication of sandwich panels via induction welding. This method enabled low-temperature joining, thereby reducing the risk of facesheet deconsolidation and core crushing—critical considerations for aerospace and automotive components where high strength, lightweight design, and weldability are paramount.

Beyond transportation and electronics, PEEK composites have also found applications in the nuclear industry. Wu et al. (2020) [70] explored 3D-printed boron carbide-PEEK composites for neutron radiation shielding and observed notable performance enhancements following appropriate heat treatment. These composites also exhibited increased tensile and flexural strength compared to conventional polyethylene-based shielding materials. In automotive engineering, PEEK is being positioned as a potential substitute for metals such as aluminum, brass, bronze, and titanium, primarily due to its superior strength-to-weight ratio and excellent resistance to grease, oil, acids, and other automotive fluids [71,72].

As research advances, PEEK continues to gain traction across a wide array of industries. Emerging applications are being explored in sectors such as textiles, oil and gas, and solar energy [73,74,75]. While both pure and composite forms of PEEK exhibit promising capabilities, many of these applications remain in the research and development phase. Continued studies are necessary to fully validate their performance and scalability in real-world environments.

## 4. Properties of PEEK

PEEK offers several advantageous attributes, positioning it as a promising alternative to traditional biomaterials [1]. Notably, its elastic modulus closely resembles that of the human bone, making it a suitable substitute for metallic biomaterials such as titanium. In addition, PEEK is radiolucent, resulting in fewer artifacts during magnetic resonance imaging [61]. However, despite these benefits, PEEK’s inherent bioinertness and low surface energy nature challenge its ability to fully replace traditional biomaterials in orthopedic and orthodontic applications [60]. To overcome these limitations, recent advancements in additive manufacturing have significantly improved the microstructure and tribological properties of PEEK, expanding its potential applications [76]. These improvements have enabled the creation of patient-specific implants, making PEEK an attractive option for load-bearing implants and orthopedic devices.

Nonetheless, the adoption of PEEK is influenced not only by its superior mechanical and biocompatible properties but also by its cost and scalability compared to traditional biomaterials such as titanium alloys or stainless steel. PEEK is a high-performance thermoplastic with a relatively higher raw material cost. For instance, medical-grade PEEK costs approximately USD 500–USD 800 per kilogram, which is substantially higher than the cost of titanium alloy powders (around USD 200–USD 400 per kilogram) or bulk stainless steel [2,77]. However, the processing and manufacturing costs must also be considered. While metals require subtractive machining, complex multi-step forming, or expensive powder bed fusion processes, PEEK can be additively manufactured via fused filament fabrication (FFF) or laser sintering with relatively simpler setups, lower energy consumption, and reduced post-processing needs [4]. Additionally, the additive manufacturing of PEEK allows for the production of patient-specific implants with minimal material wastage, which may offset its higher material cost in small-batch or customized implant production [4].

From a scalability perspective, the traditional injection molding of PEEK remains cost-effective for the mass production of standard devices, whereas additive manufacturing provides a scalable route for low-volume, customized, or complex porous structures. Recent studies suggest that as additive manufacturing technology for PEEK matures, the per-part cost is expected to decrease due to improvements in process efficiency and printer accessibility [4].

Overall, while PEEK remains a more expensive biomaterial in raw material cost compared to metals, its scalability through additive manufacturing for customized medical devices, along with reduced machining requirements and lighter weight, can make it a cost-effective option in specific biomedical applications where design complexity and biocompatibility outweigh raw material expenses. Table 1 illustrates the key properties of PEEK in comparison to titanium and UHMWPE.

### 4.1. Thermal and Radiative Properties

PEEK’s radiation resistance and thermal stability enhance its tolerance to high temperatures and gamma radiation, making it suitable for healthcare applications, particularly in the sterilization of medical devices [17,79]. In surgical settings, sterilization processes are routinely used to disinfect surgical devices and implants. These procedures expose materials to high temperatures, pressure, and humidity, which can compromise the dimensional stability and functional integrity of the implants. Therefore, considerable research has focused on evaluating the thermal resilience of medical-grade PEEK under sterilization conditions [79]. For example, Sharma et al. (2023) investigated the effects of steam sterilization on the dimensional characteristics of 3D-printed PEEK cranial implants and found that the material maintained high dimensional accuracy post-sterilization [43]. Similarly, Chai et al. (2022) [80] examined the effects of gamma irradiation on PEEK’s surface and internal structure, focusing on its tribological behavior. Their results showed that gamma-irradiated PEEK exhibited lower dynamic friction and increased surface oxidation, beneficial for lubrication applications. However, higher irradiation doses led to surface degradation, increased wear rate, and the formation of defects like micropores and microcracks, which negatively impact the surface hardness and wear resistance. These results underscore the need for further research to optimize irradiation doses and sterilization methods, ensuring that the mechanical and tribological properties or PEEK are preserved for critical biomedical applications [81].

### 4.2. Porosity

PEEK is widely recognized for its biocompatibility, which underpins its growing use in biomedical applications. However, a major limitation remains its relatively poor osseointegration, particularly in orthopedic and dental contexts. To address this, research has increasingly focused on strategies to enhance bone implant integration without compromising mechanical integrity.

A commonly investigated approach involves introducing controlled porosity into PEEK structures. By increasing surface area and promoting cellular activity, porous architectures can improve biological fixation. Evans et al. (2015) proposed that tailoring porosity could enhance bone ingrowth while preserving the material’s load-bearing capacity [82]. Building on this, Gummadi et al. (2022) [7] fabricated porous PEEK scaffolds using fused filament fabrication (FFF), evaluating pore sizes from 100 µm to 600 µm. Their findings identified 300 µm as the optimal pore size for orthopedic load-bearing applications, with larger pores resulting in diminished mechanical strength.

**Figure 6:**
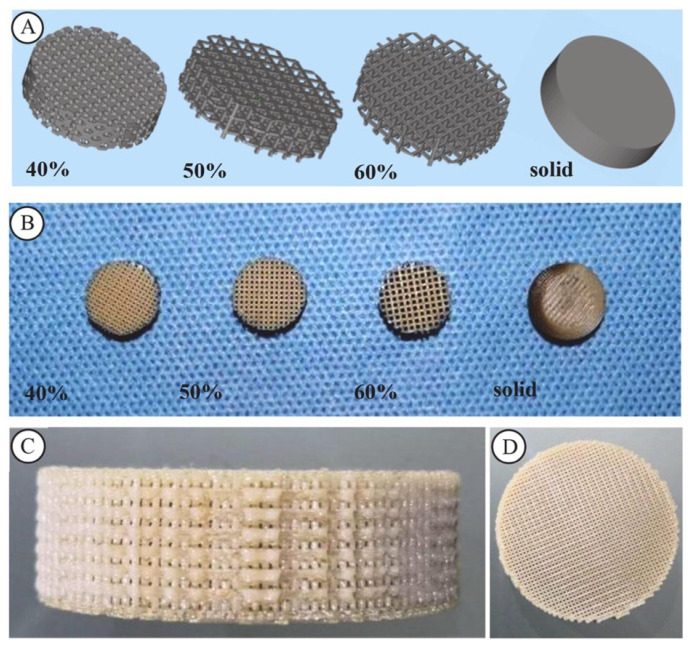
The modified porous PEEK implants. (**A**) The CAD of the modified implants; (**B**) the modified PEEK implants: from left to right: 40%, 50%, 60%, and solid; (**C**) side view of the implant; (**D**) front view of the implant. Reprinted from Wong et al. [83]. Licensed under CC BY-NC-ND 4.0 (https://www.sciencedirect.com/science/article/pii/S1751616121002241 (accessed on 13 July 2025)).

Similarly, Wong et al. (2021) [83] employed fused deposition modeling (FDM) to create cranium PEEK implants with varying porosities, shown in Figure 6. In vivo experiments indicated no adverse biological responses. Notably, implants with 40% porosity demonstrated optimal bone compatibility and osseointegration. These structures supported the adhesion of platelets, osteocytes, and monocytes, while the increased surface roughness further encouraged bone growth.

Beyond geometric design, printing parameters also significantly influence the porosity and mechanical performance of PEEK implants. Basgul et al. (2018) [45] examined lumbar fusion cages printed at varying speeds and assessed their compressive, shear, and torsional properties. Their results highlighted an optimal print speed between 1000 and 1500 mm/min, balancing reduced production time with mechanical integrity. Moreover, a direct correlation was observed between print speed and porosity, with faster speeds producing higher porosity.

In summary, modifying PEEK’s porosity—through both structural design and process parameters—can markedly enhance its osseointegration potential. Continued research is essential to refine printing techniques that yield porous structures without sacrificing mechanical performance. These advancements hold significant promise not only for orthopedic implants but also for applications in dental implants and tissue engineering.

### 4.3. Crystallinity

Crystallinity plays a critical role in determining the mechanical properties of PEEK. Achieving a well-ordered crystalline structure typically necessitates a controlled, gradual cooling process [84]. To enhance crystallinity in additively manufactured PEEK, numerous studies have explored post-processing treatments—particularly thermal annealing—as a means of improving both structural and mechanical performance [85].

**Figure 7:**
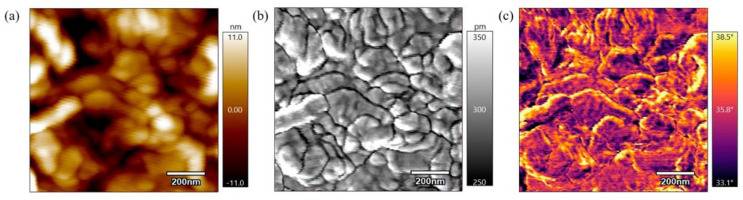
AFM scans of PEEK annealed for 6 h at 360 °C: (**a**) height, (**b**) amplitude, and (**c**) phase scans. Adapted from Adamson et al. [87]. Licensed under CC BY 4.0 (https://www.mdpi.com/2073-4360/17/6/744 (accessed on 13 July 2025)).

Zhen et al. (2023) [86] examined the effects of post-processing heat treatment on 3D-printed PEEK components. Their approach involved preheating an oven to a target temperature, placing the PEEK parts inside for a designated period, and subsequently cooling them in ambient air. The results indicated that higher annealing temperatures and extended exposure times significantly improved both crystallinity and mechanical strength. In a related study, Adamson and Eslami (2025) [87] applied a similar annealing process to five 3D-printed PEEK specimens to assess changes in surface morphology and crystallization. Their results revealed that samples treated at 360 °C for six hours exhibited enhanced crystallinity and more uniform surface textures. Atomic force microscopy (AFM) imaging (Figure 7) confirmed the formation of larger and more distinct grain boundaries, indicating improved molecular ordering and surface stability.

While these studies highlight the value of post-processing heat treatments in promoting crystallinity and mechanical integrity, further investigations are warranted. Specifically, there is a growing need to evaluate how other processing parameters—such as raster angle, ambient temperature, nozzle temperature, and printing speed—influence the crystalline morphology and microstructural characteristics of PEEK components [88,89].

Shirani Bidabadi et al. (2024) [90] employed an alternative approach to enhance the crystallization of PEEK by using a hybrid nanomaterial system composed of cellulose nano-crystals (CNC) and graphene nanoplatelets (GNP). The CNC:GNP specimens were shown to be the optimal hybrid system in improving the tensile strength and modulus by altering the crystalline morphology. However, more work is required to fully understand the long-term mechanical performance and crystalline morphology of these hybrid systems.

Beyond heat treatment, other post-processing techniques such as surface polishing, metal coating, and UV curing have been explored to enhance the properties of polymer-based materials [91]. Overall, post-processing 3D-printed PEEK remains under investigation, and further research is essential to develop a more comprehensive understanding.

### 4.4. Fatigue and Fracture Behavior of PEEK

Another important aspect of processing PEEK is understanding its fatigue behavior, which is critical when designing implants subjected to cyclic loading [92]. Previous research has investigated the stress-life fatigue behavior and fracture characteristics of notched PEEK under cyclic loading, including the percentage of the lifetime spent in crack initiation versus propagation. The results showed that increasing the notch severity and cyclic stress level reduced the number of cycles to failure, with a significant portion of the lifetime spent in the crack initiation phase [93].

**Figure 8:**
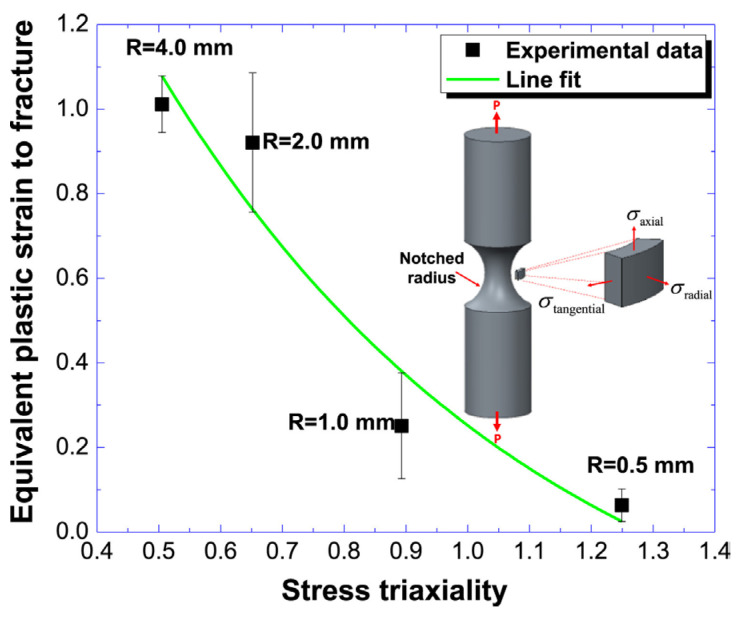
Equivalent plastic strain to fracture with the change in stress triaxiality. Adapted from Chen et al. [94]. Licensed under CC BY 4.0 (https://www.sciencedirect.com/science/article/pii/S1751616116302430 (accessed on 13 July 2025)).

Another study focused on the impact of stress concentrations, such as notches and irregular shapes, on the fracture behavior of PEEK implants. The stress–strain curve of different notched radii, illustrated in Figure 8, revealed that increasing the radius from 0.5 mm to 4.0 mm reduced stress triaxiality. The study concluded that implant designs with reduced stress concentrations—such as smaller notches, stress-relieving grooves, and narrower notches—can enhance performance under cyclic loading [94].

Rendas et al. (2023) [95] studied the fatigue behavior of 3D-printed PEEK under tension–tension cyclic loading at varying stress levels ranging from 75% to 95% of its ultimate tensile strength. The material exhibited an above-average fatigue strength of about 65 MPa (~75% of ultimate tensile strength), highlighting PEEK’s ability to withstand high cyclic stress levels close to its ultimate tensile strength before failure. However, fracture surface analysis revealed crack formations during printing, suggesting that adjusting the printing parameters can significantly impact fatigue strength. Table 2 summarizes key advances in the fatigue and fracture behavior of PEEK in load-bearing applications.

Although significant research has investigated the fatigue behavior of PEEK, further studies are needed to fully understand its fatigue and fracture behaviors under cyclic loading conditions similar to those in the human body, including long-term tension, compression, and testing in environments that replicate physiological conditions. Similarly, it is crucial to identify the optimal printing parameters to enhance the performance of PEEK implants produced using additive manufacturing.

### 4.5. Biocompatibility and Physiological Toxicity

PEEK has been widely investigated and utilized in biomedical applications due to its excellent biocompatibility and negligible physiological toxicity. As a bioinert polymer, PEEK does not elicit significant adverse reactions when implanted in the human body. This is a critical advantage for orthopedic, spinal, and craniofacial implants where long-term stability and minimal immune response are required. Multiple in vitro and in vivo studies have demonstrated that PEEK exhibits minimal cytotoxicity, does not induce inflammatory responses, and maintains stable interactions with surrounding tissues [2]. For instance, Kurtz and Devine (2007) reported that PEEK implants showed similar or better cellular adhesion and proliferation compared to metallic implants when used in spinal cages and orthopedic devices [2]. Similarly, comparative studies have shown that PEEK exhibits cytotoxicity and pro-inflammatory responses similar to traditional dental implant materials such as titanium alloys and zirconia [78].

Additionally, PEEK is chemically stable within the physiological environment and does not release toxic degradation products under normal sterilization and implantation conditions [96]. Its radiolucency allows for clear post-operative imaging without artifacts, unlike metallic implants, providing additional clinical safety and diagnostic benefits [2]. Overall, the FDA approval of PEEK for various implantable medical devices underscores its demonstrated safety, biocompatibility, and favorable performance in long-term biomedical applications.

However, PEEK is inherently bioinert rather than bioactive, meaning that it does not naturally bond to bone. To enhance osseointegration, osteoconductivity, and cellular interactions, surface modifications such as plasma treatment, chemical etching, or hydroxyapatite coating are commonly employed [97]. Section 5 will explore these surface modification approaches in greater detail.

## 5. Limitations and Modifications of PEEK

PEEK continues to gain attention in various fields due to its favorable attributes in different settings. However, ongoing research has identified several limitations that constrain its application in some areas. Researchers are actively exploring solutions, primarily through surface modification, to address these challenges. The following subsections review key limitations and the efforts made to overcome them.

### 5.1. Hydrophilicity

**Figure 9:**
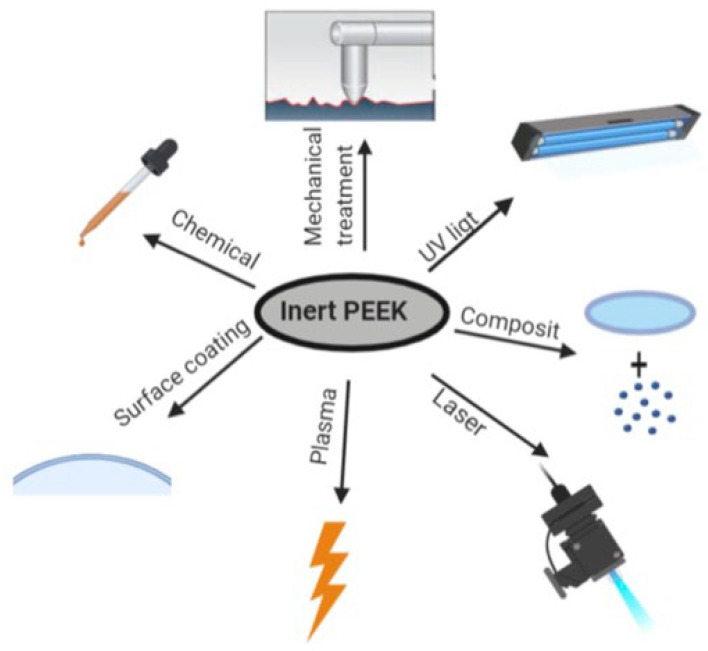
Various surface modification methods to improve the properties of PEEK. Adapted from Omrani et al. [100]. Licensed under CC BY 4.0 (https://biointerfaceresearch.com/wp-content/uploads/2020/01/20695837102132140.pdf (accessed on 13 July 2025)).

The inherent hydrophobicity of untreated PEEK surfaces significantly limits their use in medical implants by hindering cell and tissue adhesion, potentially leading to implant failure. Surface modification techniques—such as surface coating, surface roughening, plasma treatment, chemical treatment, and laser modification—have been employed to improve PEEK wettability and bioactivity [98,99,100]. Key modification techniques are illustrated in Figure 9.

One study reviewed various surface modification techniques, such as sandblasting, corona treatment, oxygen plasma treatment, argon plasma treatments, laser irradiation, and acid etching, to enhance the surface properties of copper-coated PEEK films. Although no single optimal technique was identified, each has its distinct advantages and drawbacks. Overall, these treatments enhanced the bonding strength, surface roughness, and oxygen-to-carbon (O/C) ratio and reduced the contact angle, all of which contribute to better cell integration [101].

Wang et al. (2022) also reviewed strategies to improve the adhesive properties of PEEK dental implants, concluding promising adhesion performance but emphasizing the need for further clinical trials to evaluate the long-term outcomes [102]. Despite extensive effort, there remains a gap in identifying the optimal approach to improve PEEK wettability [100]. In the following subsections are several promising surface modification techniques previously explored in the literature.

#### 5.1.1. Physical Modifications

Physical surface modification has shown promising results in enhancing the surface wettability and tissue adhesion of PEEK. Several studies have focused on modifying PEEK’s surface characteristics by altering its architecture, surface morphology, and stiffness. Microscale and nanoscale surface modifications—such as sandblasting, plasma treatment, and accelerated neutral atom beam (ANAB) technology—have been shown to promote osteointegration by changing the structure and surface morphology of PEEK [103].

One study evaluated the influence of sandblasting on the wettability of PEEK disks, indicating that increased surface roughness led to a decrease in the contact angle, suggesting enhanced wettability [104]. In another in vitro study, the surface of PEEK copings on underlying dentin abutments was modified using sandblasting and UV radiation, revealing that the combination of sandblasting and UV radiation produced the highest retention strength, while the UV radiation-only specimens exhibited the lowest strength [105].

Similarly, Porrelli et al. (2021) demonstrated that combining sandblasting with air-plasma treatment significantly enhances the wettability and surface roughness of PEEK samples [106]. Other techniques, such as corona-discharge treatment and (ANAB) technology, have also been shown to reduce the contact angle and enhance the hydrophilicity of PEEK surfaces [107,108]. However, there is limited literature concerning these techniques, and further investigation is needed to fully understand their impact on long-term in vivo applications.

While individual approaches have shown promising results, combining multiple techniques appears more effective in enhancing bioactivity and promoting bone ingrowth on PEEK surfaces [109]. As a result, ongoing research continues to explore these combinations to optimize PEEK performance across various biomedical domains.

#### 5.1.2. Chemical Modifications

Combining chemical and physical surface modification techniques has been shown to further enhance PEEK’s surface properties. Zhao et al. (2020) [110] proposed a two-step strategy that mimics the natural structure of joint cartilage by anchoring an acrylic acid-co-acryl amide hydrogel layer onto the surface of PEEK via UV-initiated polymerization. The hydrogel-coated PEEK substrate was then immersed in a ferric nitrate solution to promote cross-linkage between the iron ions and the carboxyl groups in the hydrogel, transforming the hydrophobic PEEK surface into a hydrophilic, low-friction surface with self-healing properties. Another promising technique explored by Luo et al. (2023) [111] involved femtosecond laser (FSL) treatment followed by hydroxylation, aimed at promoting osteogenic activity in PEEK implants in a fast, simple, and environmentally friendly approach. This approach increased surface roughness and significantly enhanced surface hydrophilicity, promoting cell proliferation and osteointegration.

**Figure 10:**
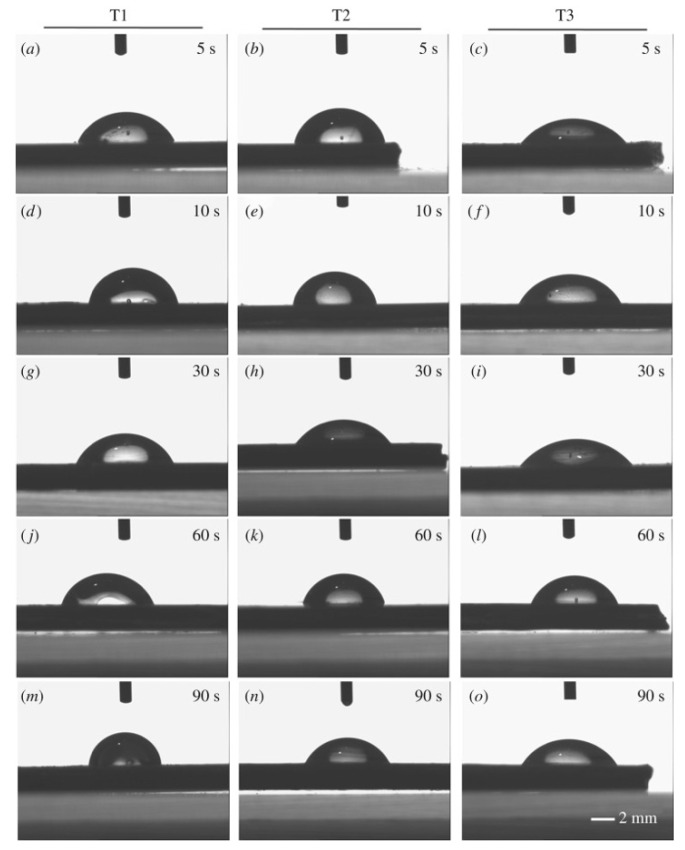
Water contact angle on PEEK surfaces modified by different sulfonation procedures and reaction durations, (**a**,**d**,**g**,**j**,**m**) Treatment 1. (**b**,**e**,**h**,**k**,**n**) Treatment 2. (**c**,**f**,**i**,**l**,**o**) Treatment 3. (**a**–**c**): 5 s, (**d**–**f**): 10 s, (**g**–**i**): 30 s, (**j**–**l**): 60 s, (**m**–**o**): 90 s. Adapted from Wang et al. [112]. Licensed under CC BY 4.0 (https://royalsocietypublishing.org/doi/10.1098/rsif.2018.0955 (accessed on 13 July 2025)).

Wang et al. (2019) [112] developed an optimal fast ambient temperature sulfonation treatment to PEEK surface hydrophilicity. Three treatment methods were tested: Treatment 1 involved sulfonation with sulfuric acid, Treatment 2 included post-sulfonation polishing with soft laboratory tissue, and Treatment 3 involved sulfonation followed by immersion in 6 wt% NaOH. The results showed that a 30 s sulfonation time with Treatment 3 was the most effective in achieving the highest hydrophilicity, as illustrated in Figure 10.

Similarly, dos Santos et al. (2021) demonstrated that sulfonation with sulfuric acid and piranha solution reduced PEEK’s water contact angle, promoted cell adhesion and proliferation, and maintained cytocompatibility, with no significant differences in cell viability compared to untreated PEEK [113].

In conclusion, the various surface modification techniques yielded different results, emphasizing the need to choose the right method—or combination of methods—based on the intended application. Table 3 summarizes these techniques and their respective performances.

### 5.2. Bioactivity

Despite its excellent mechanical strength and biocompatibility, polyetheretherketone (PEEK) is inherently bioinert, which limits its surface energy, protein adsorption, and cellular adhesion—ultimately resulting in poor osseointegration [103,114]. To overcome these limitations, researchers have investigated various surface modification techniques, including the application of bioactive coatings such as hydroxyapatite (HA), calcium silicate, and bioglass [115]. For instance, Johansson et al. (2018) [116] employed a spin coating method to apply HA onto PEEK implants, which were then implanted into the tibias and femurs of rabbits. Their findings demonstrated significantly improved bone formation and osseointegration, particularly during the early stages of healing (2–12 weeks). Similarly, Hong et al. (2018) used a dip coating technique to deposit a bioactive glass-chitosan (BG-CH) composite on PEEK surfaces, resulting in enhanced cell adhesion, proliferation, and differentiation—demonstrating its potential for orthopedic applications [117].

However, a major concern with coated implants is the potential for coating delamination over time. The detachment of surface layers may release particles into the surrounding tissue, potentially compromising implant stability and leading to adverse biological reactions [118]. To address this issue, alternative strategies such as scaffold fabrication using additive manufacturing have been explored. Roskies et al. (2016) [119] utilized selective laser sintering (SLS) to fabricate porous PEEK scaffolds and seeded them with bone marrow stromal cells (BMSCs) and adipose-derived mesenchymal stem cells (ADSCs) harvested from Sprague Dawley rats. The study revealed that these tissue-engineered PEEK scaffolds successfully supported mesenchymal cell growth and integration, thereby enhancing osteointegration—particularly for applications in craniofacial reconstruction. Given the broad array of surface modification approaches and their associated limitations, further research is essential to refine or eliminate suboptimal methods and to establish the most effective techniques for enhancing the bioactivity of PEEK implants without compromising their mechanical integrity.

## 6. Clinical Translation of PEEK Implants

### 6.1. Regulatory Considerations

The clinical translation of PEEK-based implants in the United States is governed primarily by the U.S. Food and Drug Administration (FDA) device classification and review processes. Since the first PEEK spinal and orthopedic implants received 510 (k) clearance in the late 1990s, most off-the-shelf PEEK devices leverage that pathway by demonstrating substantial equivalence to predicate devices [2,120]. Novel PEEK constructs lacking suitable predicates must instead undergo the more rigorous Premarket Approval (PMA) process, which requires comprehensive bench testing, animal studies, and clinical trials to establish safety and effectiveness. For patient-specific, 3D-printed implants manufactured in limited quantities, the Custom Device Exemption (CDE) may apply; devices under CDE must still comply with Good Manufacturing Practices (GMP) as defined in 21 CFR 820 and submit annual usage reports to the FDA [121]. Moreover, the FDA’s guidance on the additive manufacturing of medical devices emphasizes the need for validated process controls, full lot traceability, and rigorous post-build testing critical considerations given PEEK’s high processing temperatures and sensitivity to print parameters [122].

Despite these regulatory avenues, several practical challenges persist in bringing 3D-printed PEEK implants to the market. First, ensuring reproducibility and dimensional accuracy across multiple prints demands robust in-process monitoring and strict quality-control protocols. Second, although PEEK’s high melting point generally permits autoclave sterilization, each new geometry must undergo sterilization validation, comparing steam or gamma radiation, to confirm that no dimensional distortion or polymer embrittlement occurs. Finally, any novel surface treatments or bioactive coatings applied to PEEK implants necessitate biocompatibility testing in accordance with ISO 10993-1:2018 [123] to rule out cytotoxicity, sensitization, or adverse inflammatory responses, even when the base polymer has established safety profiles.

### 6.2. Long-Term Clinical Performance and Failure Modes

PEEK has demonstrated promising long-term outcomes in various biomedical applications, particularly in spinal, craniofacial, and joint implants. Clinical studies have reported high success rates for PEEK-based implants, such as a 93.7% success rate and a relatively low complication rate of 15.4% in cranioplasty procedures using 3D-printed PEEK implants [124]. Despite these encouraging results, several failure modes have been identified that warrant further investigation. One of the most significant challenges is PEEK’s inherent bioinertness, which can lead to poor osseointegration and implant loosening over time [125]. This limitation has prompted the development of surface modification techniques, including plasma treatment, sulfonation, and hydroxyapatite coatings, to enhance bone implant integration. Another concern is wear particle generation, particularly in joint replacements, where PEEK and its composites may release debris that can trigger inflammatory responses. Although carbon fiber-reinforced PEEK (CFR-PEEK) has shown improved wear resistance, the biological impact of long-term exposure to wear particles remains under scrutiny. Additionally, fatigue and fracture under cyclic loading are critical considerations for load-bearing implants. Studies have shown that optimizing implant geometry and additive manufacturing parameters can significantly improve fatigue resistance. Coating delamination is another potential failure mode, especially in implants with bioactive surface layers, as it may compromise implant stability and lead to adverse tissue reactions. Overall, while PEEK exhibits excellent mechanical and chemical stability, continued research is essential to address these long-term performance challenges and to validate its reliability across diverse clinical scenarios.

## 7. Conclusions

Polyetheretherketone (PEEK) is widely acknowledged as a high-performance polymer that combines exceptional mechanical, thermal, and chemical properties, positioning it as a highly versatile material across diverse industries. Over recent years, extensive research has sought to optimize PEEK’s structural, surface, and biological characteristics. This comprehensive review highlights recent advancements in enhancing PEEK and its composites, with a focus on biomedical applications, where precise control over material properties can profoundly influence patient outcomes.

PEEK’s favorable elastic modulus, radiolucency, chemical inertness, and fatigue resistance has led to its increasing adoption as an alternative to conventional biomaterials like titanium alloys and UHMWPE. It is widely used in clinical applications, including spinal cages, craniomaxillofacial reconstruction, joint replacements, dental prostheses, and rib implants, underscoring its versatility and clinical promise. Additionally, additive manufacturing techniques has further propelled the fabrication of patient-specific implants with controlled porosity and complex geometries, enabling tissue integration and load-bearing capabilities.

Despite these notable strides, PEEK’s bioinert nature and hydrophobic surface continue to pose significant challenges, limiting protein adsorption and cellular adhesion essential for osteointegration and long-term implant success. Surface modification strategies—including plasma treatment, sandblasting, acid etching, laser irradiation, and bioactive coatings such as hydroxyapatite and bioglass—have demonstrated promising results in enhancing PEEK’s bioactivity. However, these modifications must balance improved biological response with mechanical strength and stability, as issues like coating delamination and particle debris highlight the need for durable surface treatments that maintain performance under physiological conditions.

Additionally, the mechanical performance of PEEK in additive manufacturing remains highly sensitive to processing parameters such as printing speed, nozzle temperature, raster angle, and post-processing heat treatments. These factors significantly influence crystallinity, porosity, and microstructural morphology, which directly affect mechanical strength, fatigue resistance, and durability. While annealing and heat treatment protocols have improved structural properties, standardized processing guidelines are needed to optimize mechanical properties without compromising efficiency or introducing defects.

Another critical avenue lies in the development of PEEK composites, particularly carbon fiber-reinforced PEEK (CF/PEEK) and boron carbide reinforced PEEK, which enhances mechanical performance with functional properties such as electromagnetic wave absorption and radiation shielding. These composites expand PEEK’s application potential beyond biomedicine into aerospace, automotive, electronics, and nuclear industries. However, they also introduce manufacturing and performance challenges that require systematic investigations into interfacial bonding, thermal stability, and long-term behavior under operational stresses.

Looking ahead, additive manufacturing is poised to further revolutionize the fabrication of PEEK-based components. Its ability to produce highly customized, multi-material, and hierarchical structures opens exciting opportunities for personalized medicine, lightweight structural components, and multifunctional devices. Nevertheless, translating laboratory-scale innovations into clinical and industrial practice requires overcoming barriers related to reproducibility, scalability, regulatory approval, long-term clinical performance, and cost-effectiveness. Interdisciplinary collaboration among material scientists, biomedical engineers, clinicians, and manufacturers will be essential to address these challenges.

In conclusion, while PEEK demonstrates remarkable potential and versatility, further advancements are needed to understand the relationships between processing parameters, microstructure, mechanical behavior, and biological interactions. Expanding upon the promising findings related to increased porosity, future studies should prioritize the investigation of PEEK’s mechanical behavior at elevated porosity levels, specifically ranging from 50% to 80%. Additionally, significant research should prioritize the development of tailored manufacturing and modification techniques for specific applications, alongside comprehensive long-term in vivo evaluations to ensure safety and efficacy. By addressing current limitations, PEEK is well positioned to become a cornerstone material in the next generation of high-performance, biocompatible, and multifunctional devices.

## Figures and Tables

**Table 1:** Comparison of key properties of PEEK, titanium, and UHMWPE.

References	Property	PEEK	Titanium	UHMWPE
[2,20,47,60]	Elasticity Modulus (GPa)	3–4	102–113	0.8–1.6
[2,14,60]	Tensile Strength (MPa)	80–97	954–976	20–49
[2]	Elongation at break (ductility)	~30%	-	-
[2]	Flexural Strength (MPa)	~140–160	-	-
[50,54]	Wear Resistance	Good	Excellent	Excellent
[1,10,60,61]	Biocompatibility	Excellent	Excellent	Good
[1,2,10,47,56]	Radiolucency	Radiolucent	Radiopaque	Radiolucent
[1,5,47]	Osteointegration	Poor	Good	Poor
[1,78]	Cytotoxicity	Low	Low	Moderate
[1,2]	Raw Material Cost	High	Moderate	Low
[1,2]	Manufacturing Cost	Moderate	High	Low

**Table 2:** Summary of key studies on the fatigue and fracture behavior of PEEK.

Reference	Focus	Findings	Conclusion
[92]	Understanding the fatigue behavior of PEEK and its composites in implant devices subjected to repeated loading.	PEEK composites, such as HA/PEEK materials, exhibit promising fatigue resistance properties suitable for biomedical applications.	Highlighted the promising future of PEEK composites in biomedical implants and stressed the need for further research into the development of fatigue damage in these materials.
[93]	Investigating the stress-life fatigue behavior and fracture characteristics of notched PEEK specimens.	More severe notch geometries and higher cyclic stress levels significantly reduced the number of cycles to failure. Majority of the fatigue lifetime was spent in the crack initiation phase.	Emphasized the importance of considering design-related stress concentrations when developing PEEK-based implants undergoing cyclic loading.
[94]	Examining the fracture behavior of PEEK specimens with varying notch radii under different stress triaxialities.	Smaller notch radii resulted in higher stress triaxiality. Designs with reduced stress concentrations demonstrated improved performance under cyclic loading conditions.	Highlighted stress triaxiality as a key design parameter for optimizing the performance of notched PEEK implants.
[95]	Investigating the high-cycle fatigue behavior of 3D-printed PEEK specimens subjected to stress-controlled tension–tension cyclic loading, highlighting the significance of cyclic loading in load-bearing implant design.	Three-dimensionally printed PEEK endured high cyclic stress levels up to 75% of its ultimate tensile strength before failure. Fracture surface analysis revealed that crack initiation occurred in voids formed during the printing process.	Highlighted PEEK’s potential for load-bearing applications. Emphasized the need for further research to understand the correlation between printing parameters and fatigue behavior.

**Table 3:** Summary of various PEEK surface modification techniques.

Reference.	Surface Modification Technique	Summary
[101,104,105]	Sandblasting/Blasting	Increased surface roughness without significantly altering the chemical composition of PEEK. Increased wettability and adhesion.
[101,107]	Corona treatment	Increased surface polarity and O/C ratio, improving adhesion and wettability. However, it decreased surface roughness.
[101]	Oxygen plasma treatment	Significantly increased the O/C ratio, enhancing adhesion and wettability. However, it decreased surface roughness.
[101]	Argon plasma treatment	Enhanced surface adhesion without increasing oxygen content. Also reduced surface roughness.
[101,105,111]	Laser treatment	Reduced surface roughness and contact angle without oxygen content. Enhanced surface hydrophilicity. May introduce surface degradation
[101]	UV radiation	Increased oxygen content on the surface PEEK, enhancing wettability and bond strength.
[101]	Chemical treatment	Strongly increased the O/C ratio and surface roughness, significantly improving adhesion.
[110]	Hydrogel-coated PEEK	Improved surface wettability and lubrication. Demonstrated self-healing properties, and reduced wear in implants.
[112,113]	Sulfonation	Improved hydrophilicity by significantly reducing the water contact angle. Created a nano-porous PEEK surface for enhanced adhesion and bioactivity.

## Data Availability

No new data were created or analyzed in this study. Data sharing is not applicable to this article.
